# Two rare cases of benign struma ovarii with malignant recurrence

**DOI:** 10.1530/EDM-23-0122

**Published:** 2024-04-12

**Authors:** Kanella Kantreva, Stavroula A Paschou, Katerina Stefanaki, Kanella Pappa, Paraskevi Kazakou, Dionysios Vrachnis, Evangelia Kavoura, Kitty Pavlakis, Eirini Giovannopoulou, Konstantinos Lathouras, Maria Alevizaki, Katerina Saltiki

**Affiliations:** 1 Endocrine Unit and Diabetes Center, Department of Clinical Therapeutics, Alexandra Hospital, School of Medicine, National and Kapodistrian University of Athens, Athens, Greece; 2 Pathology Department, IASO Hospital, Athens, Greece; 3 Department of Gynecological Oncology, IASO Hospital, Athens, Greece

**Keywords:** Adult, Female, White, Greece, Ovaries, Endocrine-related cancer, Tumours and neoplasia, Gynaecological endocrinology, Error in diagnosis/pitfalls and caveats, April, 2024

## Abstract

**Summary:**

Struma ovarii is an ovarian teratoma that comprises 2–5% of all ovarian teratomas. Malignant transformation of struma ovarii occurs in less than 5% of all cases, and metastatic disease is even rarer. We report two cases initially diagnosed with benign struma ovarii that presented malignant transformation, specifically highly differentiated follicular carcinoma of the ovary (HDFCO), some years after the first diagnosis. Case 1 concerns a 37-year-old female featuring HDFCO of the right ovary with multiple metastatic foci, who was diagnosed with benign struma ovarii 14 years ago. Case 2 concerns a 26-year-old female diagnosed with HDFCO of the left ovary. This patient was initially diagnosed with benign struma ovarii 6 years ago that recurred 4 years after the diagnosis. Both patients were treated with surgery, adjunctive total thyroidectomy, and radioactive iodine (^131^I) therapy.

**Learning points:**

- Malignant transformation of struma ovarii is very rare (<5%).
- Diagnosis of HDFCO without extra ovarian dissemination is difficult due to the resemblance of its histological appearance with normal thyroid tissue.
- There is no consensus on the postoperative treatment of malignant struma ovarii (MSO). Clinical and histological features of MSO should be assessed for the postoperative treatment decisions.
- TSH suppression and thyroglobulin level measurements are necessary for patient follow-up.

## Background

Struma ovarii is an ovarian teratoma defined by the presence of thyroid tissue, that comprises more than 50% of the overall mass. It constitutes 2−5% of ovarian teratomas and 1% of all ovarian tumors (1). The majority of struma ovarii cases are benign. Malignant transformation of struma ovarii is very rare (less than 5% of all cases), and metastatic disease occurs even more rarely (2). The majority of malignant struma ovarii (MSO) cases account for papillary (70%) or follicular carcinomas (2). Highly differentiated follicular carcinoma arising from the ovary (HDFCO) is a rare version of struma ovarii, often accompanied by extra ovarian dissemination in the peritoneal cavity (also referred as peritoneal strumosis), and sometimes by systemic dissemination (3, 4). The differential diagnosis between HDFCO at an early stage with no extra ovarian spread and benign struma ovarii is very difficult because the histological features of HDFCO resemble those of normal thyroid tissue (4, 5). We report two cases of HDFCO struma ovarii, both diagnosed as benign struma ovarii several years ago, and treated at our institution.

## Case presentation 1

A 37-year-old Caucasian premenopausal female was referred to our institution from the Gynaecological Department for further evaluation of a possible secretory activity of a neoplasm located in the right ovary. She was admitted to the hospital because of a right ovarian mass, detected by ultrasound, measuring 9.96 cm. Fourteen years ago, in 2004, the patient underwent a right ovarian cystectomy, with the histopathological diagnosis of a benign struma ovarii (‘benign mature cystic ovarian teratoma, which contained thyroid tissue’). At the age of 4, she had a history of a kidney tumor (nephroblastoma), which was operated on and treated with external radiation and chemotherapy. Moreover, she had a history of polycystic ovary syndrome and diabetes type 2, managed with metformin and vildagliptin. She complained of menstrual irregularity. She had five uncomplicated births. Her family history was positive for tumorigenesis (brother with a diagnosis of sarcoma with pulmonary metastases, sister with a history of hysterectomy because of leiomyomas, and mother with goiter and adrenal adenomas).

### Investigation

**Figure 1:**
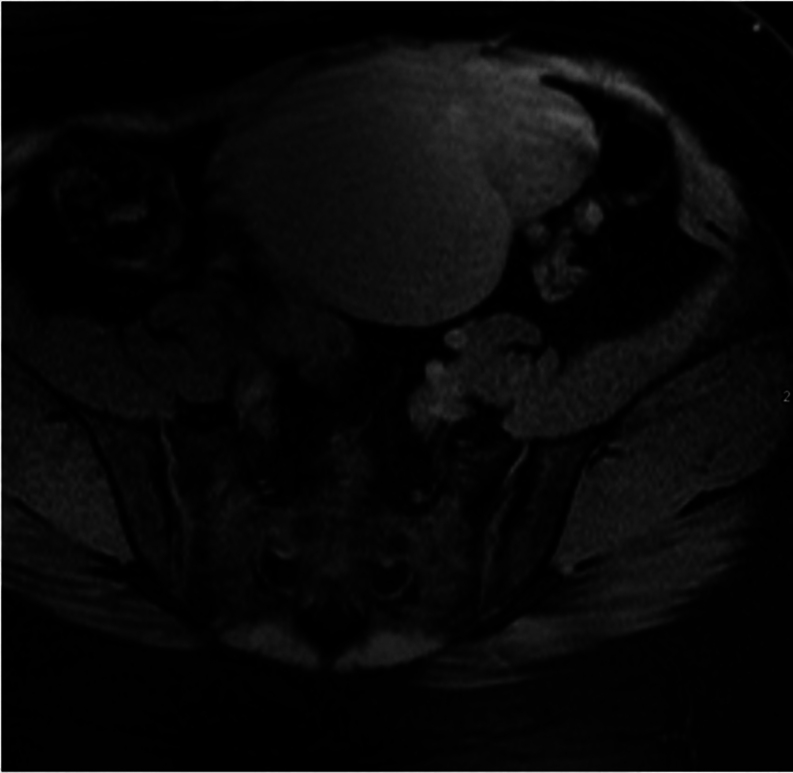
Pelvic MRI mass within the right pelvis that arose from the right adnexa (case 1).

From the clinical examination, the patient was obese (BMI: 37.1) with a palpable node in the left lobe of the thyroid and a palpable mass in the right pelvis. She had mild hypertrichosis, evaluated as grades 3–4 on the Ferriman–Gallway score. The patient was euthyroid, and apart from an abnormal glycemic profile (Hba1c: 7%, glucose: 260 mg/dL), all other tests including AFP, LDH, CEA, Ca19-9, Ca-125, and androgens were within the normal range. Abdomen imaging with CT showed nodular formations in the left ovary and peritoneum and a mass within the right pelvis, measuring 12.8 × 9.7 cm, originating from the right adnexa. Pelvic MRI with contrast confirmed the lesion, as well as nodular formations in the left ovary, peritoneum, and perisigmoid fat (Fig. 1). Total hysterectomy, appendectomy, and omentectomy were performed. Histological examination revealed a well-differentiated follicular carcinoma of the thyroid in the context of struma ovarii in both ovaries, appendix, and peritoneum (‘peritoneal strumosis’).

### Treatment

Due to the aggressive behavior of the neoplasm, total thyroidectomy and ablation with radioactive ^131^I (RAI) were recommended. After thyroidectomy, the final pathology of the thyroid revealed a microscopic non-invasive follicular thyroid neoplasm with papillary-like nuclear features (NIFTP) with a maximal diameter of 0.75 cm, without disruption of the capsule or invasion of capillaries. Additionally, a benign thyroid nodule measuring 16 mm was detected. The patient was treated with 60 mCi of radioactive iodine ^131^I after thyroxine withdrawal.

### Outcome and follow-up

**Figure 2:**
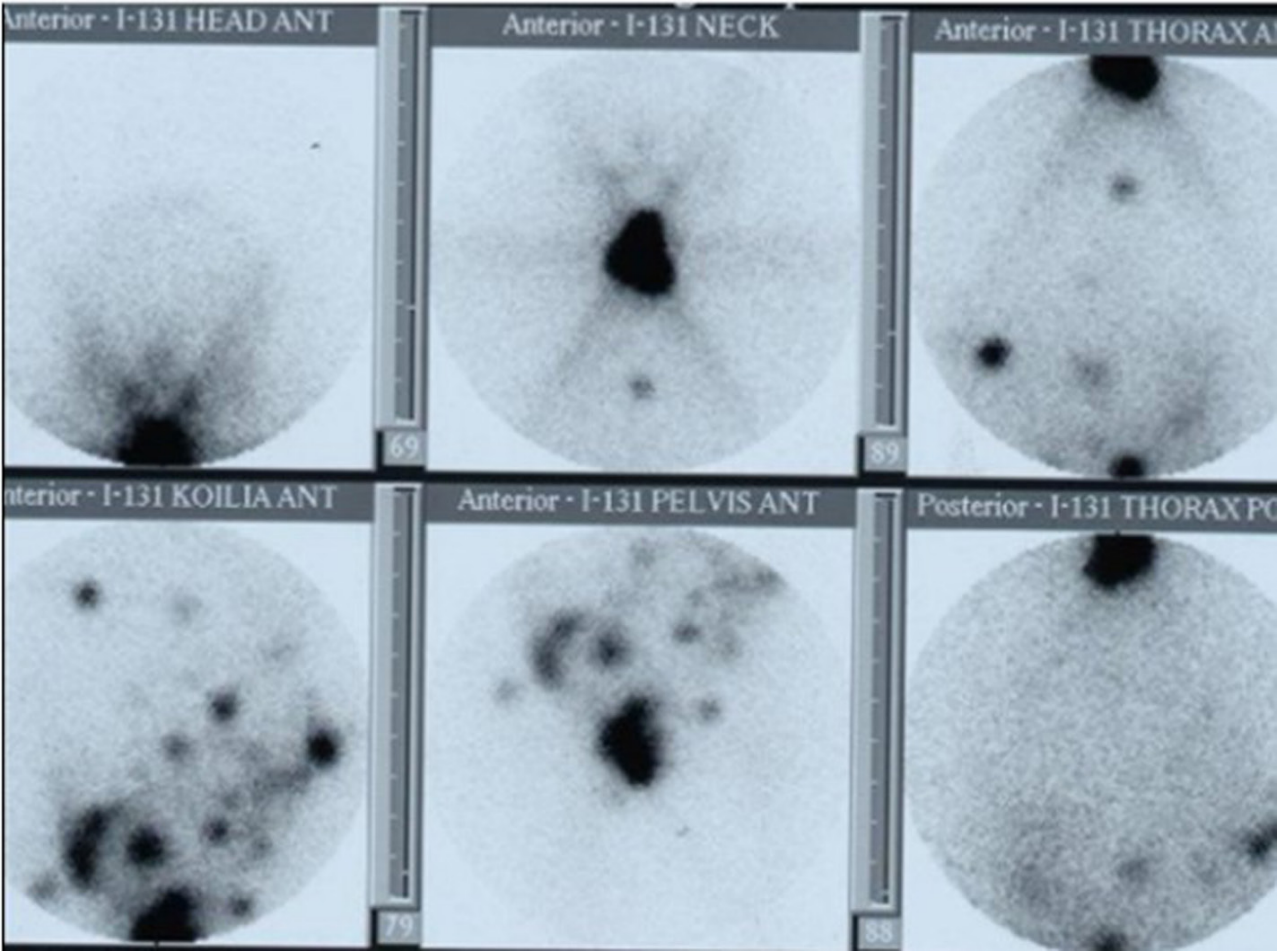
Postablative whole-body scan (after 1st RAI therapy) showed increased uptake with a star effect at the anterior cervical area and selectively increased uptake in the thorax and multiple sites in the abdomen (case 1).

The postsurgical stimulated (after withdrawal) thyroglobulin level (sTg) prior to radioiodine therapy was 144 ng/dL. After ablation, basal thyroglobulin level (bTg) was reduced to 2.1 ng/dL. The post-ablative whole-body scan (WBS) showed increased uptake with a star effect in the anterior cervical area and selectively increased uptake in the thorax and multiple sites in the abdomen (Fig. 2). After ablation, suppression therapy with levothyroxine (T4 175 µg 1 × 1) was initiated. Cervical ultrasound and post-therapeutic WBS after 9 months were normal. Additional pelvic MRI and a bone scan were negative for metastatic disease. However, a conducted recombinant human TSH (Thyrogen) test revealed increased levels of stimulated thyroglobulin (32.98 ng/dL) with negative anti-Tg (Table 1). Due to residual disease, a second radioiodine treatment with 150 mCi ^131^I was recommended. The post-ablative WBS revealed selectively increased uptake at the anatomical site of the right hip. For further evaluation, a pelvic MRI of the hip was performed, ruling out any signs of secondary metastatic disease.

**Table 1:** Tg and thyroid hormone levels at diagnosis and during follow-up (case 1).

	**TSH**(μIU/mL)	**FT4**(pmol/L)	**Tg**(ng/dL)	**Anti-Tg**(IU/mL)
Before hysterectomy	1.82	18	38	(–)
Before thyroidectomy	1.34	18.2	23.7	(–)
After thyroidectomy	5.5	16.1	25	(–)
After thyroxine withdrawal	>100	2.57	144	(–)
After 1st RAI and T4 suppression	0.053	24.1	2.1	(–)
Thyrogen test (9 months post 1st RAI)	27.9		32.98	(–)
After thyroxine withdrawal	>100	2.6	16.7	(–)
After 2nd RAI and T4 suppression (3 months)	0.026	34	0.9	(–)
Thyrogen test (1 year post 2nd RAI)	10.2		23	(–)
After 2nd RAI and T4 suppression (5 years)	0.04	25.87	0.4	(–)

One year after the second radioiodine therapy, the Thyrogen test showed persistently elevated levels of stimulated thyroglobulin (23 ng/mL) (Table 1) with negative anti-Tg, whereas imaging revealed no pathological findings. At follow-up, basal Tg levels remain stable (0.4–0.9 ng/mL – Table 1). No structural recurrence has been detected through imaging. Thus, the patient continues to be closely monitored for biochemical disease persistence.

## Case presentation 2

A 26-year-old female was referred to our institution for further evaluation after surgical removal of a mass of the left adnexa (October 2021). In March 2015, she underwent laparoscopic right ovarian cystectomy after an incidental ultrasonographic finding of a right adnexal mass. Histopathologic examination exhibited a cystic teratoma consisting mostly of thyroid tissue, confirming the diagnosis of benign struma ovarii. In March 2019, the patient presented ascites and abdominal pain. Computed tomography revealed a right ovarian cystic lesion measuring 5 cm with possible peritoneal implantations and a cystic lesion of the liver. Ascitic fluid was punctured. Ascitic fluid cytology tests exhibited the absence of cells of squamous origin. Ca-125 levels were elevated. A laparoscopic right salpingo-oophorectomy with peritoneal biopsies was performed. Histopathologic examination of the right ovary revealed a recurrence of struma ovarii. Histopathologic examination of the peritoneum revealed peritoneal inclusion cysts. The patient received no further treatment, and regular follow-up was recommended.

### Investigation

**Figure 3:**
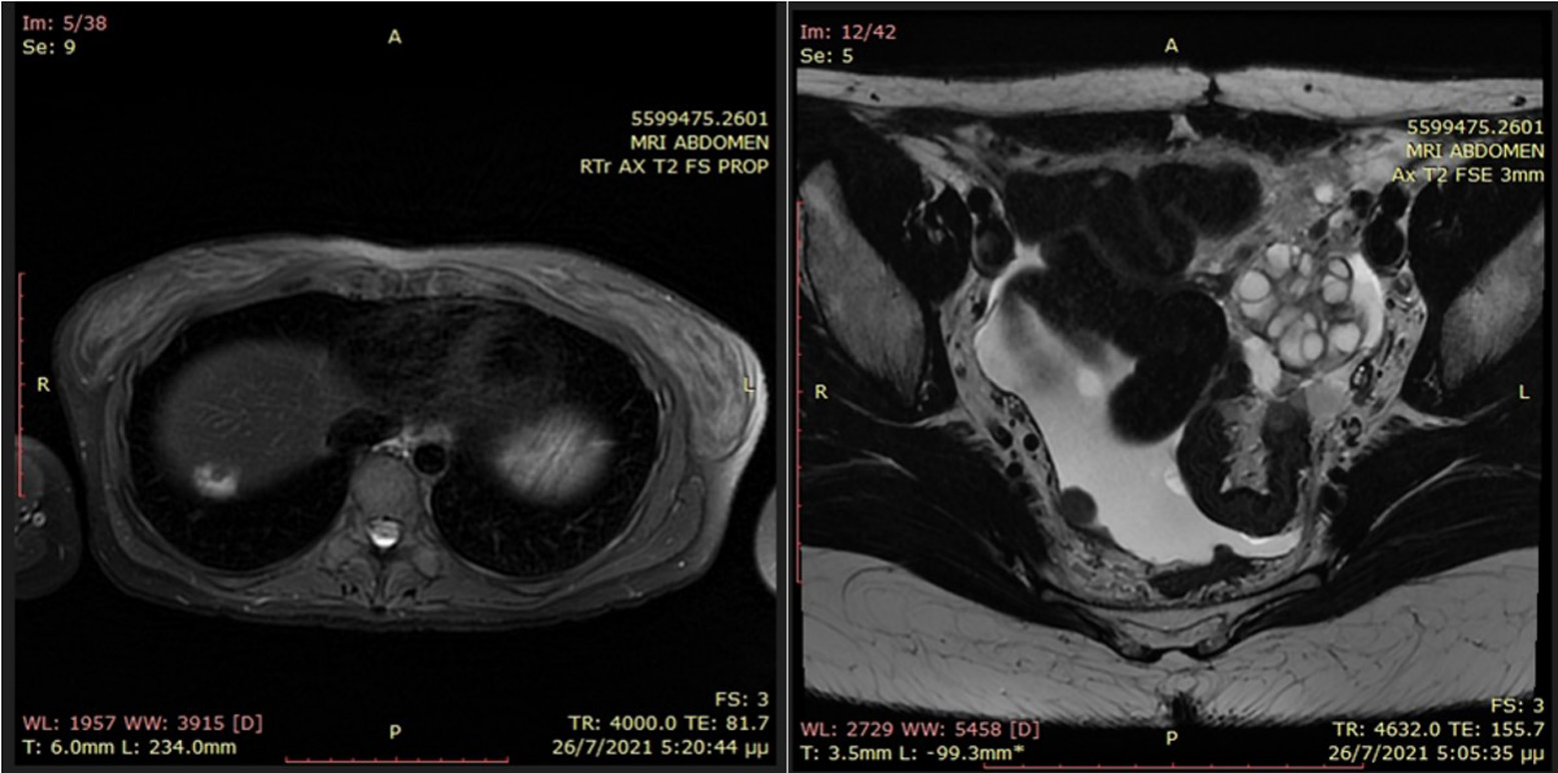
Abdomen MRI T2. (A) Lesion at the capsule of the liver (1.5 cm), below the diaphragm. (B) Mass complex at the left ovary and salpinx area (6.0 × 4.2 × 5.7 cm), with multiple cystic spaces (case 2).

**Figure 4:**
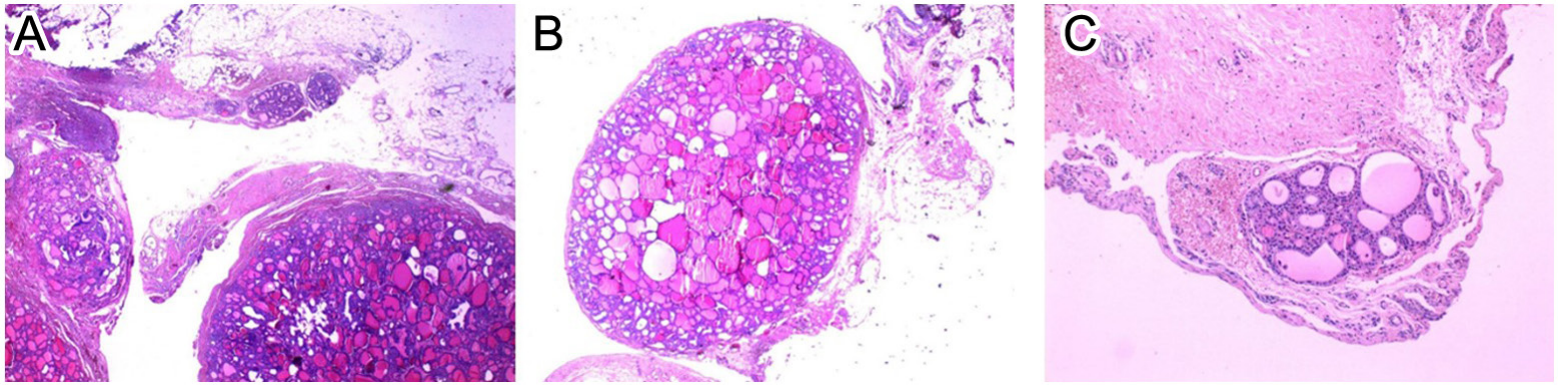
(A) Multiple omental nodules of benign-appearing thyroid tissue. (B) One single omental nodule of benign-appearing thyroid tissue. (C) One single peritoneal nodule of benign-appearing thyroid tissue (case 2).

In July 2021, the patient was admitted to the gynecological department due to abdominal pain. Pelvic MRI exhibited a mass complex at the left ovary and salpinx area (6.0 × 4.2 × 5.7 cm), intraperitoneal effusion, multiple nodules, and cystic lesions in the peritoneal cavity, as well as a heterogeneous lesion at the capsule of the liver, below the diaphragm (Fig. 3). The patient had no signs of hyperthyroidism, and thyroid hormones were normal. Thyroglobulin levels were elevated (2047 ng/mL) (Table 2). In August 2021, the patient underwent laparoscopy with a left salpingo-oophorectomy, partial omentectomy, appendicectomy, peritonectomy, and pelvic-aortic lymphadenectomy. Pathological examination exhibited a very well-differentiated follicular carcinoma (Fig. 4).

**Table 2:** Tg and thyroid hormone levels at diagnosis and during follow-up (case 2).

	**TSH**(μIU/mL)	**FT4**(pmol/L)	**Tg**(ng/mL)	**Anti-Tg**(IU/mL)
Before surgery (2021)	1.27	14.40	2047	(–)
After surgery	1.64	12.4	118	(–)
After thyroxine withdrawal	38.12	5.4	490	(–)
After first RAI and T4 suppression (4 months)	0.01	22.26	16.2	(–)
After first RAI and T4 suppression (10 months)	0.01	20.7	23	(–)
After thyroxine withdrawal	98.21	1.0	238	(–)
After second RAI and T4 suppression	0.01	19.3	10.9	(–)

### Treatment

Postoperative thyroglobulin levels were significantly lower (118 ng/mL). In October 2021, the patient underwent total thyroidectomy in terms of preparation for adjuvant radioiodine therapy. Histopathological analysis of the thyroid specimen showed nodular thyroid hyperplasia. In December 2021, the patient received 150 mCi ^131^I after thyroxine withdrawal for 4 weeks (stimulated Tg levels after thyroxine withdrawal were 490 ng/mL). After the ablation, a suppression therapy with levothyroxine was initiated (Table 2).

### Outcome and follow-up

**Figure 5:**
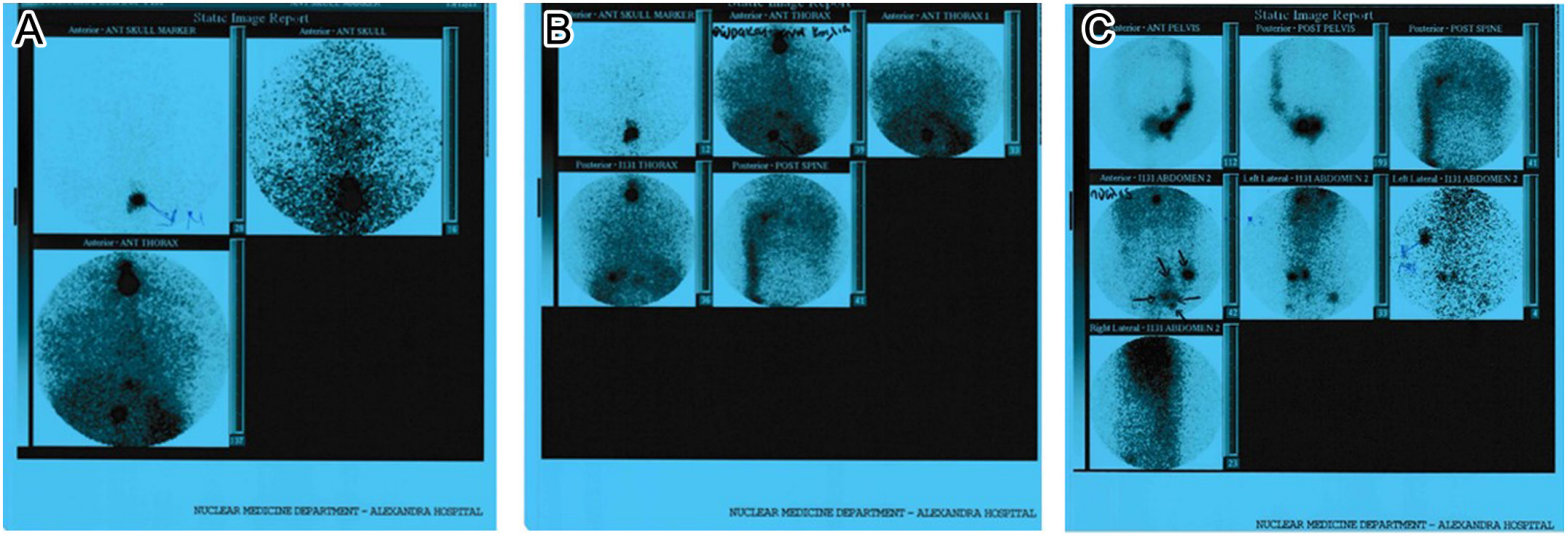
Postablative whole-body scan (after 1st RAI therapy) showed thyroid remnant uptake, uptake from multiple foci in the left pelvis, a focus below the right diaphragm, and a focus under the liver (case 2).

The postradioiodine WBS showed thyroid remnant uptake as well as uptake from multiple foci in the left pelvis, a focus below the right diaphragm, and a focus under the liver (Fig. 5). One month later, bTg was 34.1 ng/mL. Additional CT of the abdomen showed only a small effusion at the pouch of Douglas. Upon re-evaluation, 4 months later, MRI showed only postsurgical lesions, and the bTg decreased to 16.2 ng/mL (Table 2). Ten months after the first RAI administration, bTg levels increased to 23 ng/mL (Table 2), and abdominal MRI revealed a new lesion under the liver. Thus, a second RAI ablation with 180 mCi ^131^I was recommended. The postablative WBS revealed selectively increased uptake from a focus below the right diaphragm, a focus under the liver, and a focus in the left pelvis. Nine months later, bTg levels are 10.9 ng/mL (Table 1). Abdomen MRI showed two small lesions in the liver (0.6 cm and 0.3 cm) and one lesion in the left pelvis (0.7 × 0.3 cm). Due to structural and biochemical disease persistence, the patient is closely monitored with thyroglobulin assessment and imaging exams.

## Discussion

We present two cases previously diagnosed as benign struma ovarii, which recurred several years after the diagnosis with metastatic spread in the pelvis. Both histologies revealed HDFCO, and both patients were treated with RAI ablation after performing total thyroidectomy. As featured in our cases, the majority of malignant SO are asymptomatic and are discovered accidentally (41%) (1). The main symptoms, if present, are abdominal pain and a palpable abdominal mass (2). Ascites is also reported in up to one-third of SO cases, and signs of hyperthyroidism are present in 5–8% of cases (6). HDFCO at early stages with no extra ovarian dissemination presents a diagnostic challenge due to its histologic resemblance with normal thyroid tissue (4). This may be the reason that a benign teratoma may present a malignant recurrence some years after the first diagnosis.

Our cases have both shown biochemical disease persistence, and one of our patients showed additionally showed structural persistence. Thus, a long follow-up period is necessary to evaluate the rate of possible disease progression and the prognosis. In two cohorts, the overall survival rate in malignant SO was estimated at 84% at a 25-year follow-up (7, 8). In the literature, the overall recurrence rate is 21.7–35%, which is higher in patients without adjuvant therapy (2, 9). The size of the tumor, the patient’s age, and the presence of ascites have been proposed as possible risk factors for poor prognosis (8).

There is no consensus on the treatment of MSO due to the rarity of the disease. Surgery options include unilateral salpingo-oophorectomy (USO), bilateral salpingo-oophorectomy, and total abdominal hysterectomy with bilateral salpingo-oophorectomy (9). For premenopausal women who desire to preserve fertility, USO is preferred. For postmenopausal women or women who do not desire to preserve fertility, total abdominal hysterectomy with bilateral salpingo-oophorectomy is recommended (2, 7, 10).

There is no consensus on the postoperative adjuvant treatment either. The literature proposes that patients with tumor size over 2 cm, with extraovarian dissemination, and aggressive histological features should be treated with adjuvant RAI therapy (10, 11). Total thyroidectomy should be performed before RAI ablation in order to optimize ^131^I uptake from the residual malignant cells, as thyroid gland cells show preferential ^131^I uptake (12, 13).

To conclude, the early diagnosis of the rare HDFCO is significant, and post-thyroidectomy radioiodine therapy is mandatory in the majority of cases. TSH suppression and thyroglobulin level measurements are necessary for the patient’s follow-up, in conjunction with abdominal imaging. We emphasize the necessary cooperation of medical specialties in terms of multidisciplinary tumor boards at specialized referral centers for these cases.

## Declaration of interest

The authors declare that there is no conflict of interest that could be perceived as prejudicing the impartiality of these case reports. K Saltiki is on the editorial board of *Endocrinology, Diabetes & Metabolism Case Reports*. K Saltiki was not involved in the review or editorial process for this paper, on which she is listed as an author.

## Funding

The authors declare that this study did not receive any specific grant from any funding agency in the public, commercial, or not-for-profit sector.

## Patient consent

Written informed consent has been obtained from both patients for publication of the case report and accompanying images.

## Author contribution statement

K Kantreva wrote the manuscript. K Saltiki and S A Paschou revised the manuscript. K Lathouras and E Giovannopoulou performed the final surgery on patient case 2. K Pavlakis and E Kavoura performed the pathology tests on patient case 2 and provided the figures. K Stefanaki, K Pappa, P Kazakou, D Vrachnis, M Alevizaki, and K Saltiki were responsible for clinical care of the patients. All authors approved the final content of article.
